# The Sequential Application of Macroalgal Biosorbents for the Bioremediation of a Complex Industrial Effluent

**DOI:** 10.1371/journal.pone.0101309

**Published:** 2014-07-25

**Authors:** Joel T. Kidgell, Rocky de Nys, Nicholas A. Paul, David A. Roberts

**Affiliations:** MACRO – the Centre for Macroalgal Resources and Biotechnology, and School of Marine and Tropical Biology, James Cook University, Townsville, Queensland, Australia; Belgian Nuclear Research Centre SCK•CEN, Belgium

## Abstract

Fe-treated biochar and raw biochar produced from macroalgae are effective biosorbents of metalloids and metals, respectively. However, the treatment of complex effluents that contain both metalloid and metal contaminants presents a challenging scenario. We test a multiple-biosorbent approach to bioremediation using Fe-biochar and biochar to remediate both metalloids and metals from the effluent from a coal-fired power station. First, a model was derived from published data for this effluent to predict the biosorption of 21 elements by Fe-biochar and biochar. The modelled outputs were then used to design biosorption experiments using Fe-biochar and biochar, both simultaneously and in sequence, to treat effluent containing multiple contaminants in excess of water quality criteria. The waste water was produced during ash disposal at an Australian coal-fired power station. The application of Fe-biochar and biochar, either simultaneously or sequentially, resulted in a more comprehensive remediation of metalloids and metals compared to either biosorbent used individually. The most effective treatment was the sequential use of Fe-biochar to remove metalloids from the waste water, followed by biochar to remove metals. Al, Cd, Cr, Cu, Mn, Ni, Pb, Zn were reduced to the lowest concentration following the sequential application of the two biosorbents, and their final concentrations were predicted by the model. Overall, 17 of the 21 elements measured were remediated to, or below, the concentrations that were predicted by the model. Both metalloids and metals can be remediated from complex effluent using biosorbents with different characteristics but derived from a single feedstock. Furthermore, the extent of remediation can be predicted for similar effluents using additive models.

## Introduction

Coal-fired energy generation produces metal-contaminated waste water with high concentrations of regulated elements such as Al, As, Cd, Mo, Se, V and Zn. The effluent is created when water is used to wash residual ash from combustion chambers and flue stacks [1]. As these effluents are typically too contaminated to be discharged, and current treatment options are restricted by cost [2], the effluent from coal-fired power stations is often stored in large Ash Dams (ADs). However, high bioavailability and rapid bioaccumulation of toxic elements from Ash Dam Water (ADW) has resulted in cases of ecotoxicity to vertebrates in ADW catchments [3], [4] and, therefore, sustainable water treatment technologies are required. Biosorption, the use of dried biological material to passively bind contaminants from waste water, is an option for the treatment of industrial effluents [5], [6]. We have recently demonstrated that dried freshwater macroalgae are an effective feedstock for the production of biosorbents, and native species from industrial facilities can be intensively cultivated to provide biomass for biosorption [6], [7]. This circumvents one of the critical constraints to algal-based biosorption, which is sourcing a sustainable feedstock for production of biosorbents that does not compete with established markets for cultivated macroalgae [8].

Biosorption exploits charge-based interactions between dissolved elements and biosorbents. Negatively charged functional groups on the surface of dried macroalgae makes it an effective biosorbent with which to remove positively charged metal cations from solution and various macroalgal biosorbents have proven effective against a range of dissolved metals [9]–[11]. However, existing biosorption research is largely limited to synthetic effluents with only one or a few elements targeted for remediation [5], [7], [12], [13]. In reality, industrial effluents contain a myriad of coexisting elements and the complexities of treating these effluents are not replicated in experiments with synthetic solutions [14]. A continued focus on the kinetics of metal uptake from synthetic effluents by biosorbents, at the expense of empirical data on the performance of biosorbents in real-world effluents, has arguably limited the application of algal-based biosorption at scale [6], [15].

The treatment of complex effluent is difficult because the dissolved ions in the effluent have a variety of properties, and hence, affinities for biosorbents [6], [16], [17]. Macroalgae are in general effective against a range of dissolved cations and the affinity of dried macroalgae for metals (e.g. Ni^2+^) can be enhanced by converting biomass to biochar through slow pyrolysis [14], [18]. However, both dried biomass and biochar have low affinities for metalloids (e.g. SeO_4_^2−^) that co-occur with metals in industrial waste water as oxyanions [19]–[21]. An additional conversion procedure, the treatment of biochar with an iron (Fe^3+^) solution, can enhance the affinity of biochar for metalloids [22]. The resulting Fe-biochar binds a range of metalloids including Se, As and Mo at a rate that is independent of pH but also releases a suite of metals (cations), that are natural components of the biomass, back into solution in the process [14], [22]. While the release of metals during deployment of Fe-biochar for the purposes of metalloid biosorption may appear counter-productive to the aims of treating waste water, sequential application of Fe-biochar to remove metalloids and then biochar to remove metals can, in principle, treat a complex effluent far more comprehensively than if any one biosorbent was applied individually. The application of multiple biosorbents to holistically treat complex waste water through biosorption has never been empirically tested.

Here we test the applicability of a multiple biosorbent treatment technique, using Fe-biochar and biochar produced from freshwater macroalgae, to treat a complex effluent containing both metalloids and metals. We first develop a predictive model for the removal of metalloids and metals by Fe-biochar and biochar, respectively, from ADW collected from Tarong power station (Queensland, Australia). We then apply the model to predict the quantity of each biosorbent that is required to maximise the removal of metalloids and metals from ADW, accommodating for any leaching of contaminants from the biomass during the use of Fe-biochar. Finally, we produce Fe-biochar and biochar from cultivated green macroalgae *Oedogonium*, that was originally isolated from the AD of Tarong power station, and quantitatively compare the efficacy of Fe-biochar and biochar deployed simultaneously, and in sequence, to remediate metalloids and metals from the ADW.

## Materials and Methods

### Industrial effluent

This study focused on ADW from Tarong coal-fired power station in south-east Queensland, Australia (26.76°S, 151.92°E). Tarong is one of Queensland's largest coal-fired power stations with a generation capacity of 1400 MW and a 46,000 ML AD to store waste water from ash disposal processes on site. The ADW was sourced directly from the AD and transported to James Cook University (JCU), Townsville in clean plastic 1000 L Intermediate Bulk Containers (IBCs) and stored at ambient temperature in 12,000 L storage tanks until use. The ADW was collected and shipped with the permission and assistance of Stanwell Energy Corporation.

### Algal biosorbents production and preparation

*Oedogonium* sp. (Genbank: KF606974 [23]) (hereafter *Oedogonium*), was used as the feedstock for the biosorbents. *Oedogonium* is a filamentous, freshwater macroalga that is native to Tarong AD. The biomass for this study was cultivated in f/2 media in 2500 L tanks during the austral summer months (January – March) in the aquaculture facility on the Townsville campus of JCU (19.33°S, 146.76°E). Prior to experiments, 2 kg of algae was harvested from the tanks and oven dried at 60°C for 48 hours (h). The biomass was then converted into biochar by slow pyrolysis under conditions previously described [24]. Briefly, *Oedogonium* was suspended in a muffle furnace and purged with N_2_ gas at 4.0 L min^−1^ while being heated to 450°C for 1 h. A sample of the biochar was converted to Fe-biochar by soaking it in a 5% Fe^3+^ solution (diluted Sigma Aldrich 45% w/v FeCl_3_ stock solution) at a density of 25 g L^−1^ for 24 h on a shaker plate (100 rpm) at 20°C. The Fe-biochar was filtered from the FeCl_3_ solution and rinsed three times with deionized (DI) water at a rate of 20 ml g^−1^ and then dried at 60°C for 48 h.

### Derivation of predictive sorption model

A model was developed using data from a previous study with Tarong ADW [14] (Table S1) to predict the change in concentration of 21 elements after the deployment of each biosorbent. Most metal and metalloid sorption occurs within the first hour of exposure and the most effective sorption occurs when the pH of the ADW is un-manipulated (pH ∼7.1) [14]. The biosorption data collected under these conditions was used to construct the predictive model. Following 1 h of exposure of Fe-biochar or biochar to ADW the q-value (the mass of an ion [µg] adsorbed from, or released into, solution per unit of biosorbent [g]), was calculated according to Volesky (2007) [7]. The q-value was determined for each combination of biosorbent (Fe-biochar and biochar) and element (Al, As, Ba, B, etc.) to derive a model that predicted the additive effect of multiple biosorbents, applied sequentially or simultaneously, on the remediation of dissolved elements within a single effluent:
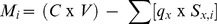



Where, M_i_ is the mass of an element in solution (µg) following treatment number *i*; C is the initial concentration of the element in solution (µg L^−1^); V is the volume of effluent solution (L); q_x_ is the q-value of the element for biosorbent, *x* (µg g^−1^); S_x,i_ is the mass (g) of biosorbent, *x*, added for treatment, *i*.

### Biosorption experiments

The aim of the biosorption experiment was to maximise the removal of oxyanionic elements (As, Mo and Se) with Fe-biochar, followed by the removal of cationic contaminants with secondary, or simultaneous, deployment of biochar. As described above, this sequential treatment strategy is logical because the targeted removal of metalloids by Fe-biochar also contributes some metals into solution. This requires a second phase of biosorption with biochar to remove existing and leached metals in ADW.

**Figure 1:**
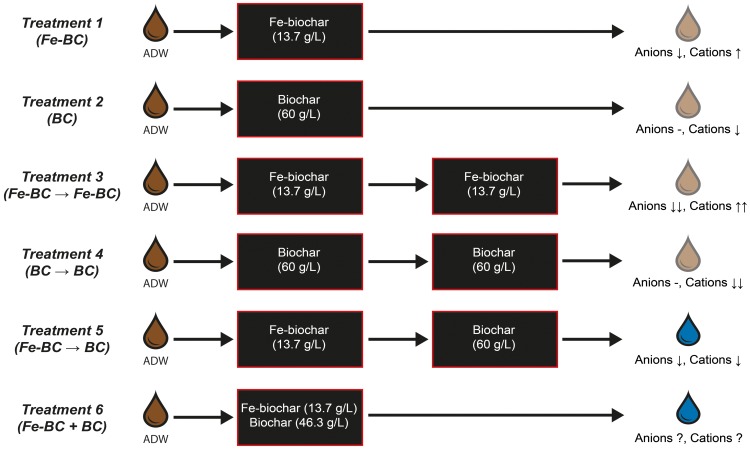
Experimental treatments and predicted changes in anion and cation concentrations in ADW. Arrows signify the direction and magnitude of predicted change (up  =  increase, down  =  decrease, dash  =  no change, ? =  unknown response). Six biosorption treatments were tested: (1) Fe-biochar (“FeBC”); (2) biochar (“BC”); (3) sequential Fe-biochar (“FeBC → FeBC”); (4) sequential biochar “BC → BC”; (5) sequential Fe-biochar and biochar (“FeBC → BC” and (6) simultaneous Fe-biochar and biochar (“Fe-BC + BC”).

In total there were 6 biosorption treatments: (1) Fe-biochar (“FeBC”); (2) biochar (“BC”); (3) sequential Fe-biochar (“FeBC → FeBC”); (4) sequential biochar (“BC → BC”); (5) sequential Fe-biochar and biochar (“FeBC → BC”); and (6) simultaneous Fe-biochar and biochar (“FeBC + BC”) (Figure 1). As the aim of the biosorption experiment was to achieve remediation of metalloids and metals, the primary treatments of interest are the sequential and simultaneous application of Fe-biochar and biochar (treatments 5 and 6 respectively, Figure 1). Treatments 1–4 were included as controls to assess the effects of Fe-biochar and biochar in isolation for comparison with the sequential and simultaneous treatments. Each treatment strategy was repeated 3 times to evaluate the model predictions.

The predictive model was used to select the loading densities of each biosorbent for the two main treatments and controls. Metalloids were targeted for removal by using a density of Fe-biochar that was predicted by the model to result in removal of Mo, the most abundant metalloid, to below ANZECC water quality criteria. The result was that Fe-biochar would be used at a density of 13.7 g L^−1^ (see further justification in results section “*Development of treatment scenarios using the predictive model”*). A limitation of the experiment was that the biochar density predicted by the model to remove all metals (110 g L^−1^), is not feasible as the maximum possible loading density of biochar or Fe-biochar was 60 g L^−1^. Therefore, all biochar treatments were loaded at a density of 60 g L^−1^ and all Fe-biochar treatments were loaded at a density of 13.7 g L^−1^. The simultaneous “Fe-BC + BC” treatment included 13.7 g L^−1^ Fe-biochar and 46.3 g L^−1^ biochar to give the maximum 60 g L^−1^ of biosorbent (Figure 1).

Each replicate consisted of a 250 ml plastic beaker with 50 ml of ADW and the appropriate mass of biosorbent. The pH of the ADW was unaltered (7.06±0.01) for all treatments. Fe-biochar or biochar (depending on treatment) was added to the ADW and placed in a shaker cabinet (Eppendorf Innova 44R) at 100 rpm in 20°C for 1 h. After 1 h, the biosorbent was separated from ADW by centrifugation (7000 rcf, 5 min), followed by two stages of filtration (75 µm nylon filter paper, then 0.45 µm syringe filter). The filtered solution was transferred to another 250 ml plastic beaker for the next treatment (biochar or Fe-biochar depending on the scenario). The process of the second sequential treatment was identical to the first, except that the mass of biosorbent was adjusted to account for any loss of solution during the separation process to ensure the same density was applied. After the second treatment the solution was processed as described and the water samples retained for analysis.

It is important to note that testing multiple treatments in the biosorption experiments provides an opportunity to validate the performance of the model when initial elemental profiles differ from those under which the model was produced. For example, after the first stage of the “BC → BC” treatment, the effluent is predicted to have low metal, but unchanged metalloid, concentration. If the model accurately predicts the performance of the second BC treatment this indicates the model is robust to variations in initial effluent characteristics. Such a finding would support the use of the model across diverse effluents with a wide range of elemental profiles.

### Elemental analysis

The concentrations of metals and light metal ions (Al, Ba, Ca, Cd, Co, Cr, Cu, Fe, K, Mn, Mg, Na, Ni, Pb, Sr, V, and Zn) and metalloids (As, B, Mo, Se) were measured using a Bruker 820-MS Inductively Coupled Plasma Mass Spectrometer (ICP-MS; Al, Ba, Cd, Co, Cr, Cu, Fe, Mn, Ni, Pb, Sr, V, Zn) or a Varian Liberty series II Inductively Coupled Plasma Optical Emissions Spectrometer (ICP-OES; Ca, K, Mg, Na). An external calibration strategy was used for both instruments, where a standard solution of 0.45 µm filtered ADW was used as the vector to calculate the concentration of elements. Collisional Reaction Interface (CRI) was used for As (CRI gas: H_2_) and V (CRI gas: He), while ^82^Se isotope was used for Se quantification, to eliminate polyatomic interferences for these elements. A 1% HCl solution was spiked with 1 ppb As, Se and V and measured three times for quality control; recovery between 98.5 and 110% indicated no significant interferences. All analyses were conducted at the Advanced Analytical Centre at JCU, Townsville.

### Data analysis

Multivariate patterns in biosorption were visualized using non-metric Multi-Dimensional Scaling (nMDS) from a Bray-Curtis similarity matrix following forth-root transformation. A Permutational Multivariate Analysis of Variance (PERMANOVA) was conducted in Primer 6.1.14 between the elements and the factor of treatment (Figure 1). Univariate analysis took the form of one-way Analysis of Variance (ANOVA), with the final concentration for each sequential treatment analysed. Data were examined for normality and homogeneity of variance using normal-probability plots of raw residuals and predicted-residual scatter plots [25]. When necessary the data were log-transformed. The PCA and ANOVA tests were conducted in Statistica (Ver. 10, Statsoft Inc.). The log-predicted final concentrations of each element from the biosorption model were plotted against the log-observed values on a scatterplot for validation of the model. A trend line of y = x was plotted to indicate where the points would be expected to lie if the observed and perfect values were in perfect agreement. Any point with a residual value > ±1 was highlighted as a deviation from the predicted concentration.

## Results

### Characteristics of ADW

Twelve (Al, As, B, Cd, Cr, Cu, Pb, Mn, Mo, Ni, Se, and Zn) of the 21 elements measured in the ADW have trigger levels established by the Australian and New Zealand Environmental Conservation Council (ANZECC, Table 1) [26]. Nine of these elements (Al, As, B, Cd, Cu, Mo, Ni, Se and Zn) were present in untreated ADW at concentrations in excess of the ANZECC trigger values for the protection of aquatic life at the 95% level (Table 1). These elements are therefore the focus of the following results and discussion.

**Table 1:** Concentration of elements in Ash Dam Water and associated ANZECC trigger values.

Element	ANZECC Trigger (µg L^−1^)	Initial Concentration [µg L^−1^ (±SE)]	
**Aluminium**	**55**	**89**	**(2.1)**
**Arsenic**	**13**	**34**	**(0.2)**
**Boron**	**370**	**3823**	**(18)**
**Cadmium**	**0.2**	**2.1**	**(0.01)**
Chromium	1	0.1	(0.02)
**Copper**	**1.4**	**2.1**	**(0.03)**
Lead	3.4	0.03	(<0.01)
Manganese	1900	0.9	(0.2)
**Molybdenum**	**34**	**3913**	**(7.2)**
**Nickel**	**11**	**38**	**(0.3)**
**Selenium**	**11**	**97**	**(0.3)**
**Zinc**	**8**	**36**	**(2.0)**
Barium	-	100	(0.3)
Calcium	-	335,333	(12,012)
Cobalt	-	0.3	(<0.01)
Iron	-	5.0	(<0.01)
Magnesium	-	99,800	(3348)
Potassium	-	44,867	(2178)
Sodium	-	446,333	(11,169)
Strontium	-	4080	(21)
Vanadium	-	982	(8.1)

Bold values exceed the ANZECC trigger value for protection of aquatic life at the 95% level.

### Development of treatment scenarios using the predictive model

**Figure 2:**
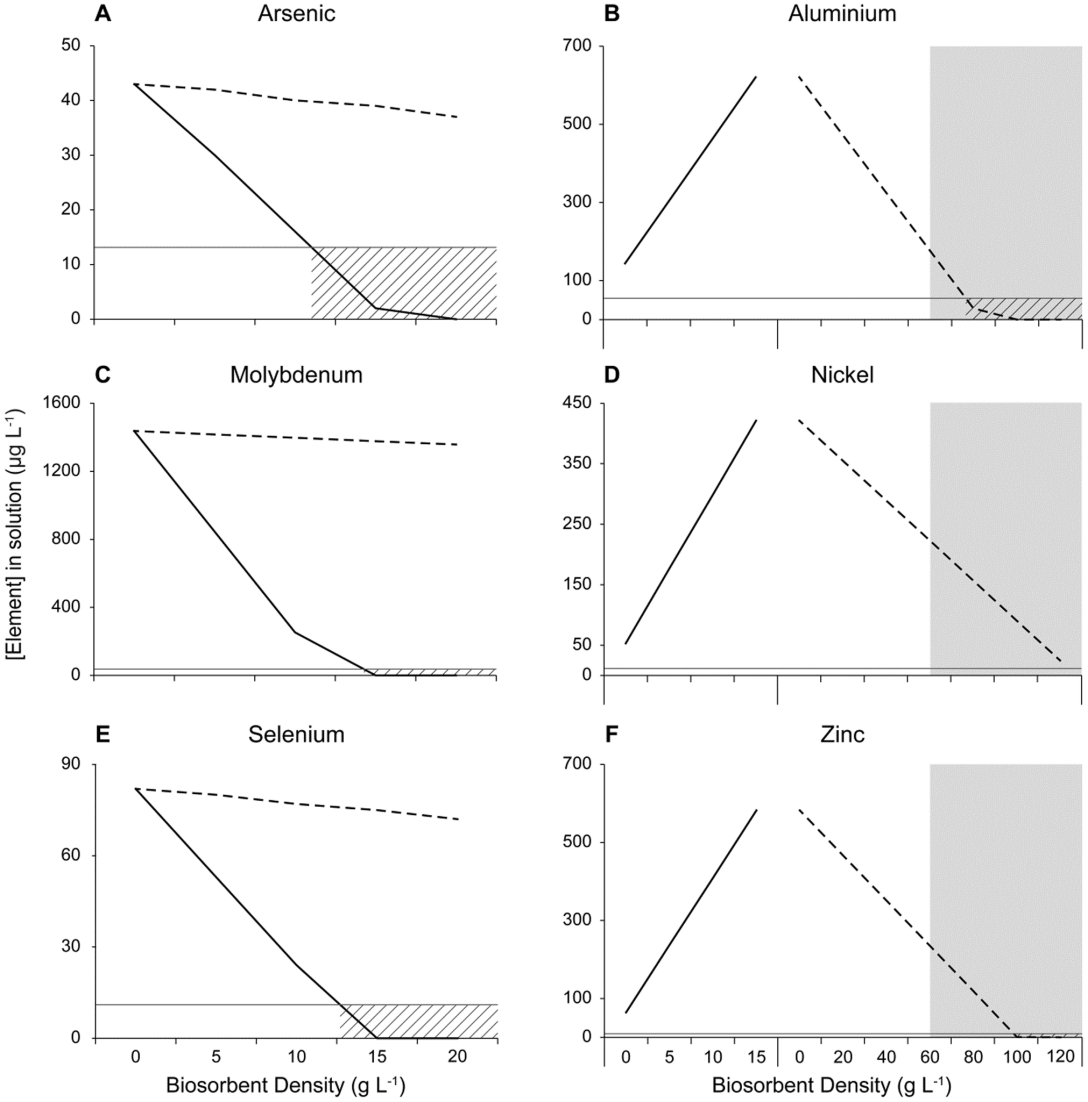
Modelled changes in concentrations of (a) As, (b) Al, (c) Mo, (d) Ni, (e) Se and (f) Zn in ADW treated with Fe-biochar and biochar (solid and dashed lines respectively). ANZECC trigger levels and stocking densities which result in reduction below the ANZECC concentration indicated by horizontal grey line and dashed area, respectively. The initial concentration of Al, Ni and Zn for the biochar treatment in plots (b), (d) and (f) are as predicted following application of 13.7 g L^−1^ Fe-biochar. The grey shaded area indicates biosorbent densities above the physical limit for experimentation (60 g L^−1^).

The model was used to predict the amount of Fe-biochar and biochar required to achieve comprehensive remediation of metalloids and metals to below ANZECC trigger values. Allowing for a 15% deviation in removal capacity, the model predicted 13.7 g L^−1^ of Fe-biochar would be sufficient to reduce the concentrations of As, Mo and Se in ADW to below the trigger values (Figure 2a, c and e). As the Fe-biochar leaches some metals into solution [14], [22], it was applied first in the sequential treatments so that the subsequent treatment of biochar could adsorb both the existing and leached metals from ADW (Figure 2b, d, f). The density of biochar required during the second treatment for removal of the metals was therefore calculated assuming 13.7 g L^−1^ Fe-biochar had been used in the first stage of treatment. A density of 110 g L^−1^ biochar was predicted for the removal of the leached and existing metals. Given physical limitations the maximum stocking density that could be achieved was 60 g L^−1^. Consequently, all biochar treatments were applied at this density (Figure 1). The simultaneous treatments were tailored to result in the maximum removal of both metalloids and metals within the physical constraints of the biochar system. Therefore, Fe-biochar was applied at a density of 13.7 g L^−1^ and biochar was added at 46.3 g L^−1^, giving the maximum stocking density of 60 g L^−1^ in treatment 6 (“FeBC +BC”, Figure 1). The simultaneous treatment (“FeBC +BC”, Figure 1) was included despite the need for different stocking densities to determine if a mixed biosorbent consisting of Fe-biochar and biochar has potential as a single-step biosorption treatment for complex effluents. As biosorption and metal leaching occurs relatively rapidly (15 min), it is possible there are rapid interactions between leached and bound elements in the simultaneous exposure that are not evident in the sequential treatments.

### Evaluation of treatment scenarios with biosorption experiments

**Figure 3:**
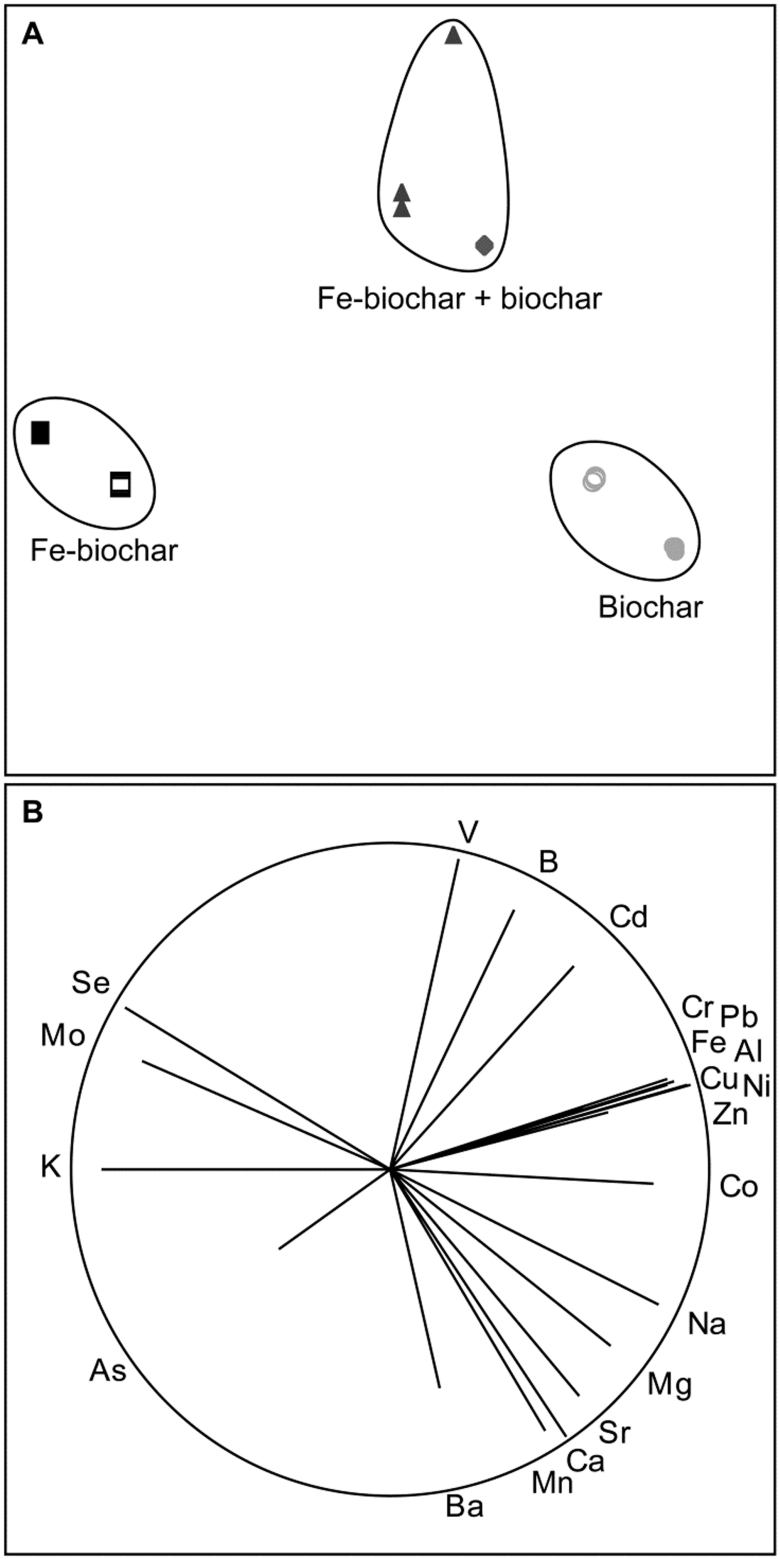
Non-metric Multi-Dimensional Scaling (nMDS) demonstrating the sorption of elements by Fe-biochar, biochar, and a combination of the two biosorbents in single and sequential treatments. (A) nMDS (Stress <0.01) with cluster analysis superimposed. Open and closed squares and circles represent single and sequential applications of Fe-biochar and biochar, respectively. Triangles represent sequential application of Fe-biochar followed by biochar and diamonds represent simultaneous application of Fe-biochar and biochar. (B) nMDS with vectors superimposed, the direction and length of indicate the strength of correlation with the treatment.

Treatment of ADW with Fe-biochar and biochar resulted in a significantly different final composition of dissolved elements than treatment with only Fe-biochar or only biochar (PERMANOVA: “treatment” pseudo-F_(3,8)_ = 79.1, p = 0.001). ADW treated with Fe-biochar (treatment 1 “FeBC” and treatment 3 “FeBC → FeBC”, Figure 1) had lower concentrations of metalloids (As, Mo and Se) than untreated ADW (Figure 3). ADW treated with biochar (treatment 2 “BC” and treatment 4 “BC → BC”, Figure 1) had lower alkali, alkaline and transition metal concentrations (Na, Mg, Sr, Ca, Cr, Pb, Fe, Al, Ni, Cu, Zn and Co) than untreated ADW (Figure 3). When ADW was treated with both Fe-biochar and biochar (treatment 5 “FeBC → BC” and treatment 6 “FeBC + BC”, Figure 1), it had a unique elemental composition compared to the other treatments, achieving significantly lower concentrations of both metalloids and metals than untreated ADW (Figure 3), with the best outcome being from the sequential treatment (treatment 5 “FeBC → BC”) which attained the lowest concentrations of metalloids and metals of all the treatment scenarios (Table 2).

**Table 2:** Change in element concentrations in ADW after exposure to Fe-biochar and biochar biosorbents (see Figure 1 for explanation of treatments).

Element	Initial concentration	Final concentration					
		FeBC	FeBC → FeBC	BC	BC → BC	FeBC → BC	FeBC +BC
Al	89±2.1	505±3.0	872±13	0.4±0.2	0.3±0.01	0.3±0.01	69±9.8
As	34±0.1	14±0.2	7.4±0.2	25±0.2	16±0.2	12±0.3	28±0.3
B	3823±18	4520±21.0	4450±4.7	4337±14	4207±22	4133±19	3650±29
Ba	100±0.3	193±5.2	623±29	94±0.4	55±1	295±13	1700±25
Ca	335,333±12,012	363,667±5683	412,333±11,703	261,667±3569	150,667±2419	423,000±2625	47,433±10,607
Cd	2.1±0.01	1.6±0.01	1.7±0.01	1.1±0.01	1.1±0.01	0.2±0.01	0.8±0.01
Co	0.3±0.01	6.8±0.2	15±0.5	0.2±0.01	0.3±0.01	1.9±0.1	5.2±0.2
Cr	0.1±0.02	13±0.8	49±9.4	2.3±0.01	3.8±0.1	3.6±0.1	1.2±0.04
Cu	2.1±0.03	20±0.3	46±1	2.1±0.02	2.3±0.1	2.9±0.1	3.3±0.1
Fe	5.0±0.01	32,833±1674	84,433±1554	8.2±2.6	5.0±0.01	5.0±0.01	281±115
K	44,867±2178	56,933±504	71,400±1576	1,000,000±9798	1,850,000±24,944	1,096,667±5443	981,000±36,003
Mg	99,800±3348	117,333±272	170,333±981	91,100±1594	74,433±542	133,000±816	14,266±981
Mn	0.9±0.2	1570±33	3880±118	0.9±0.1	1.1±0.04	2887±181	4827±60
Mo	3913±7.2	242±5.7	43±2	4597±28	4617±26	298±8.6	2300±37.4
Na	446,333±11,169	459,667±8060	507,667±3031	321,000±5312	224,000±2625	400,333±3209	446,667±18,764
Ni	38±0.2	924±35	1777±78	11.6±0.4	7.3±0.3	89±5.8	96±6.6
Pb	0.03±0.01	2.7±0.1	6.7±0.1	< LOD	< LOD	0.03±0.01	0.03±0.01
Se	97±0.3	37.3±0.2	24±0.9	112±0.7	112±0.5	40±0.4	50.4±0.8
Sr	4080±21	5413±22	5810±36	3690±45	2227±17	6343±47	5807±28
V	982±8.1	1390±12	1323±7	1333±2.7	1313±12	542±6.4	316±2.6
Zn	36±2.0	438±17	2167±28	2.5±0.1	5.7±0.2	2.5±0.01	53±3.6

All data at mean concentrations (µg L^−1^ ± S.E.).

#### a) Sorption by Fe-biochar alone (“FeBC” and “FeBC → FeBC”)

**Figure 4:**
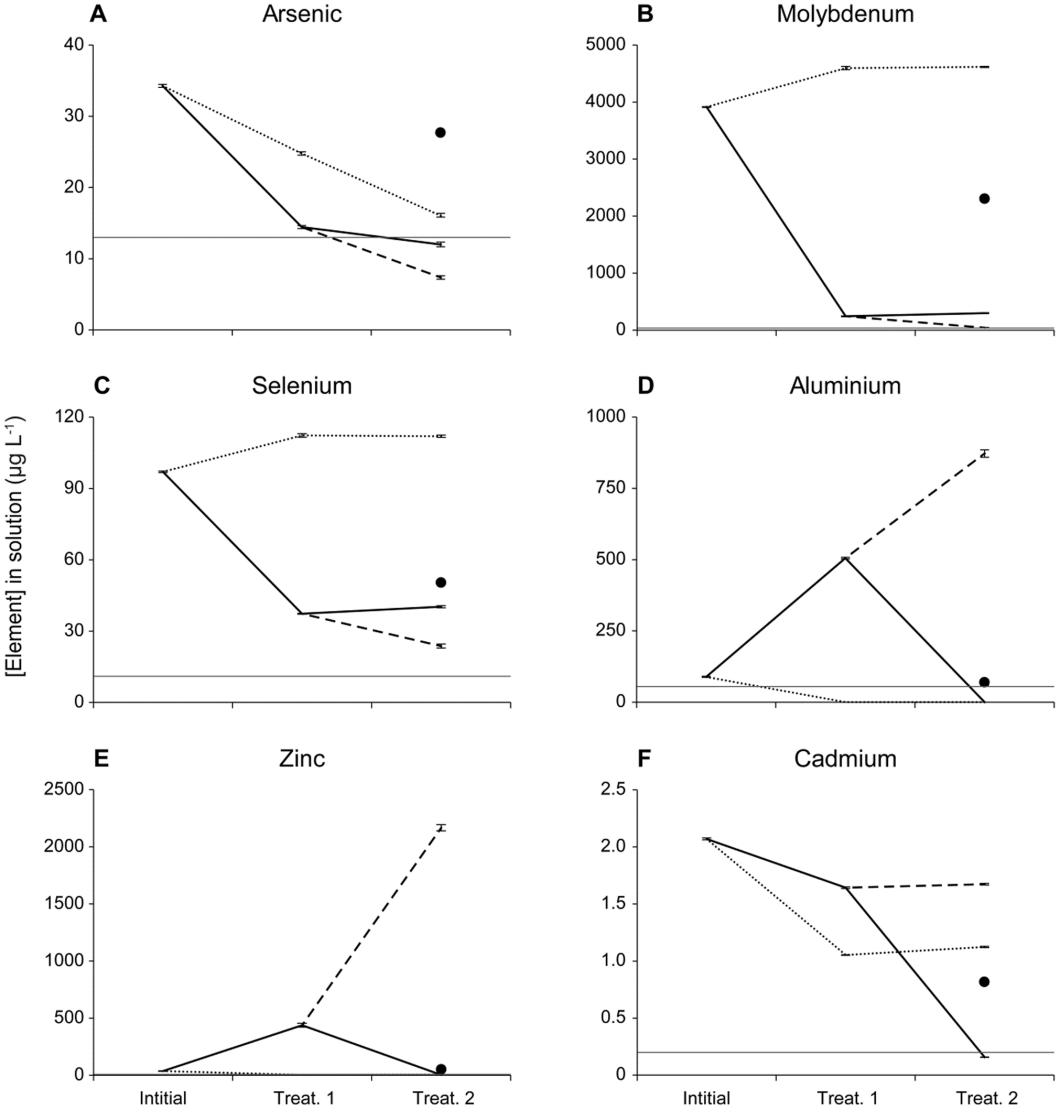
Change in solution concentration of (a) As, (b) Mo, (c) Se, (d) Al, (e) Zn, and (f) Cd in ADW after sequential exposure to Fe-biochar and biochar. The “BC”, “FeBC” and “FeBC → BC” treatments are represented as dotted, dashed and solid lines, respectively. The “FeBC + BC” treatment is represented by the circle in each panel. The “FeBC + BC” result is placed under treatment 2 to compare with the final concentrations of the other treatments. ANZECC trigger level is represented by a horizontal grey line.

The metalloids (As, Mo and Se) were removed from ADW by Fe-biochar (Figure 4a–c; Table 2). Two applications of Fe-biochar in sequence (treatment 2 “FeBC → FeBC”, Figure 1) resulted in greater removal of metalloids than any other treatment, but also resulted in the highest concentrations of dissolved metals being released (Figure 4, Figure S1). For example, Mo decreased from 3913 to 43 µg L^−1^ in ADW (Figure 4b, Table 2), while Zn increased from 36 to 2167 µg L^−1^ (Figure 4e, Table 2). The first deployment of Fe-biochar was more efficient at removing the metalloids than the second sequential deployment, which yielded lower removal rates of As, Mo and Se per unit Fe-biochar used (Figure 4a–c). Therefore, ADW exposed to “FeBC” and “FeBC → FeBC” treatments was characterised by lower metalloid and higher metal concentrations than untreated ADW (Table 2).

#### b) Sorption by biochar alone (“BC” and “BC → BC”)

Metals (Al, Cd, Cu, Mn, Ni, Pb and Zn) were removed from ADW by biochar (Figure 4d–f, Table 2). The final concentrations of Al, Cu, Mn, Ni, Pb, and Zn were lowest following the sequential biochar treatment (“BC → BC”) (Figure 4d–e, Table 2). Al, Ni and Zn were all decreased to below their trigger levels in ADW during the sequential treatment “BC → BC” (Figure 4d–e, Table 2). As was also reduced in concentration in the “BC → BC” treatment, however, the final concentration was higher than the final concentration in the “FeBC → FeBC” treatment (Figure 4a, Table 2). The change in the concentration of K was the opposite to the other metals, with substantial leaching occurring, resulting in a total increase in the concentration in ADW of over 1,800,000 µg L^−1^ in the “BC → BC” treatment (Figure S1, Table 2).

#### c) Sequential and simultaneous treatments (“FeBC → BC” and “FeBC + BC”)

The sequential treatment of ADW using Fe-biochar to remove metalloids followed by biochar to target the existing and leached metals (treatment 5 “FeBC → BC”) resulted in the most comprehensive treatment of the ADW (Table 2). As described above, metalloids were removed by the initial application of Fe-biochar while the metals, Al, Cr, Cu, Ni, Pb and Zn all leached off the biosorbent into solution (Figure 4). However, during the subsequent application of biochar, most of these metals were adsorbed to below initial concentrations (e.g. Figure 4d–e), with the exception of Cu and Ni (Table 2). Of the eight ANZECC metals (Al, Cd, Cr, Cu, Mn, Ni, Pb, Zn), four (Al, Cd, Pb, Zn) were reduced in ADW to the lowest concentrations measured across all treatments, and an additional three (Cu, Ni, Mn) were reduced to the second lowest concentrations in ADW in the “FeBC → BC” treatment (Table 2, Table S2).

When Fe-biochar and biochar were used simultaneously (treatment 6 “FeBC + BC”) there were higher final concentrations of As, Mo and Se in ADW compared to “FeBC → FeBC” and “FeBC → BC” treatments (Figure 4a–c). B was reduced to its lowest concentration in the “FeBC + BC” treatment, however, this only constituted a 4% drop from the initial concentration (Table 2, Table S2). Similarly, Cr was reduced to the lowest concentration following a treatment with “FeBC + BC” of 1.2±0.04 µg L^−1^, however, this concentration is still an order of magnitude higher than the initial concentration of Cr in ADW, 0.1±0.02 µg L^−1^ (Table 2, Table S2). Both Cu and Ni had equivalent concentrations between the treatments of “FeBC → BC” and “FeBC + BC” (Table 2, Table S2). An interesting result was the response of V, which reduced in concentration following sequential and simultaneous treatments, but increased slightly in concentration with individual treatments of biochar and Fe-biochar (Figure S1).

### Evaluation of the model

**Figure 5:**
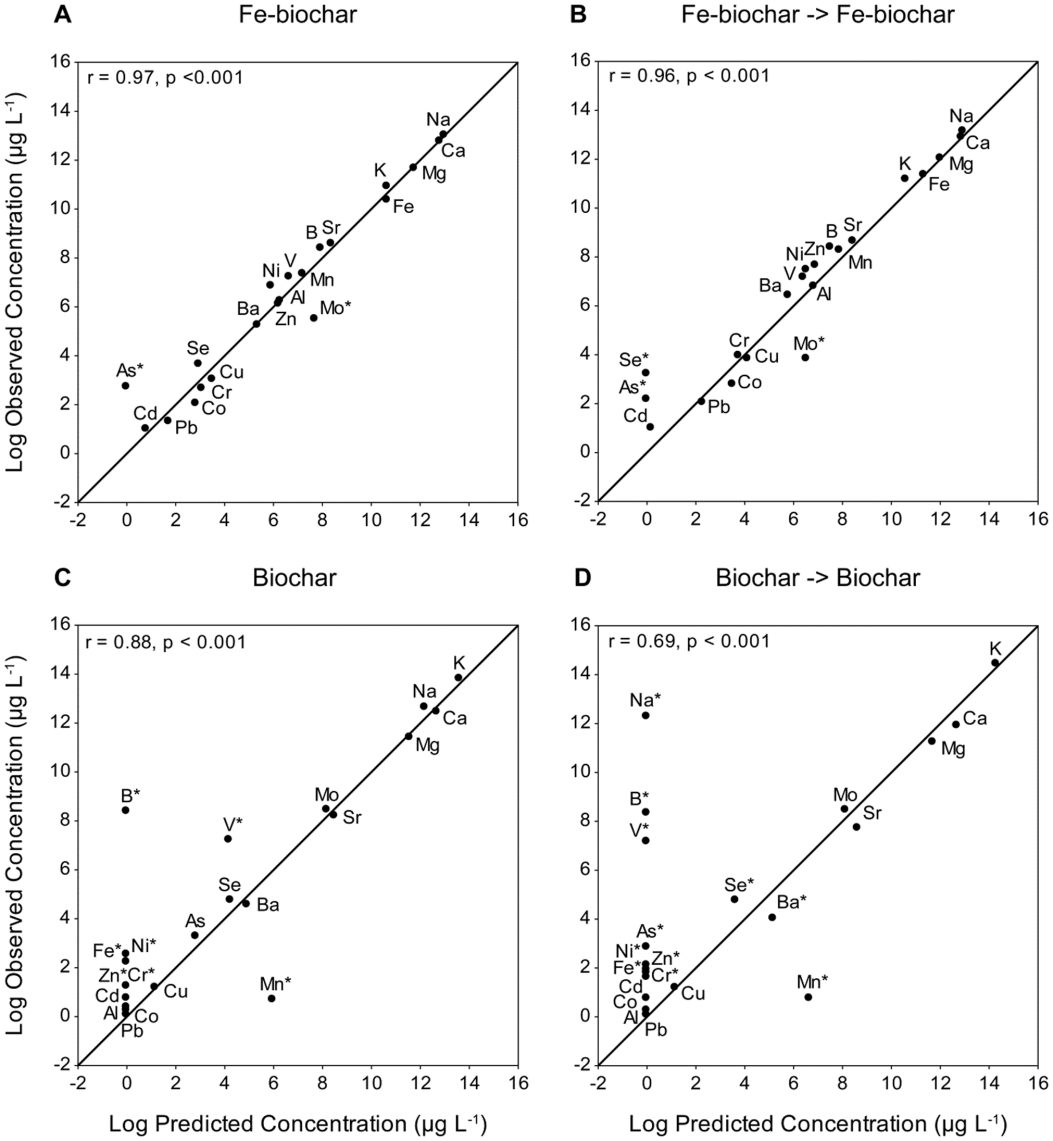
Scatterplots of predicted vs. observed concentrations of elements in ADW (µg L^−1^) following exposure to (a) “FeBC”, (b) “FeBC → FeBC”, (c) “BC”, and (d) “BC → BC” treatments. The data have been log transformed. The plotted line shows the expected relationship if all observed values are identical to predicted values. Points falling above the line were overestimated by the model (higher than expected final concentrations). Points falling below the line were underestimated by the model (lower than expected final concentrations). Asterisks indicate elements with a residual of greater than ±1.

The final concentrations of elements following the “FeBC” and FeBC → FeBC” treatments were close to those predicted by the model (r = 0.97 and 0.96 respectively, Figure 5a–b). The treatment of ADW with Fe-biochar delivered greater than expected reductions in Mo, but was less effective than predicted for As (in both single and sequential treatments) and Se (in sequential treatments) (Figure 5a and b). The model was slightly less accurate for predicting the concentrations following “BC” and “BC → BC” treatments, as demonstrated by the weaker correlations between predicted and observed concentrations (r = 0.88 and 0.69 respectively, Figure 5c, d). Despite the weaker correlation, the concentration of 14 of the 21 elements was accurately predicted for a single application of biochar (Figure 5c). The treatment of ADW in the “BC” treatment resulted in higher than predicted concentrations of B and V and lower than expected concentrations of Mn (Figure 5c). The deviations from model prediction were compounded in the “BC → BC” treatment as 11 elements (As, B, Ba, Cr, Fe, Mn, Na, Ni, Se, V & Zn) out of the 21 had residual values > ±1. Na had the greatest deviation from expected concentration (Figure 5d; Table 2). B, V and Mn had similar deviations from predicted concentrations in the “BC → BC” treatment (Figure 5d).

**Figure 6:**
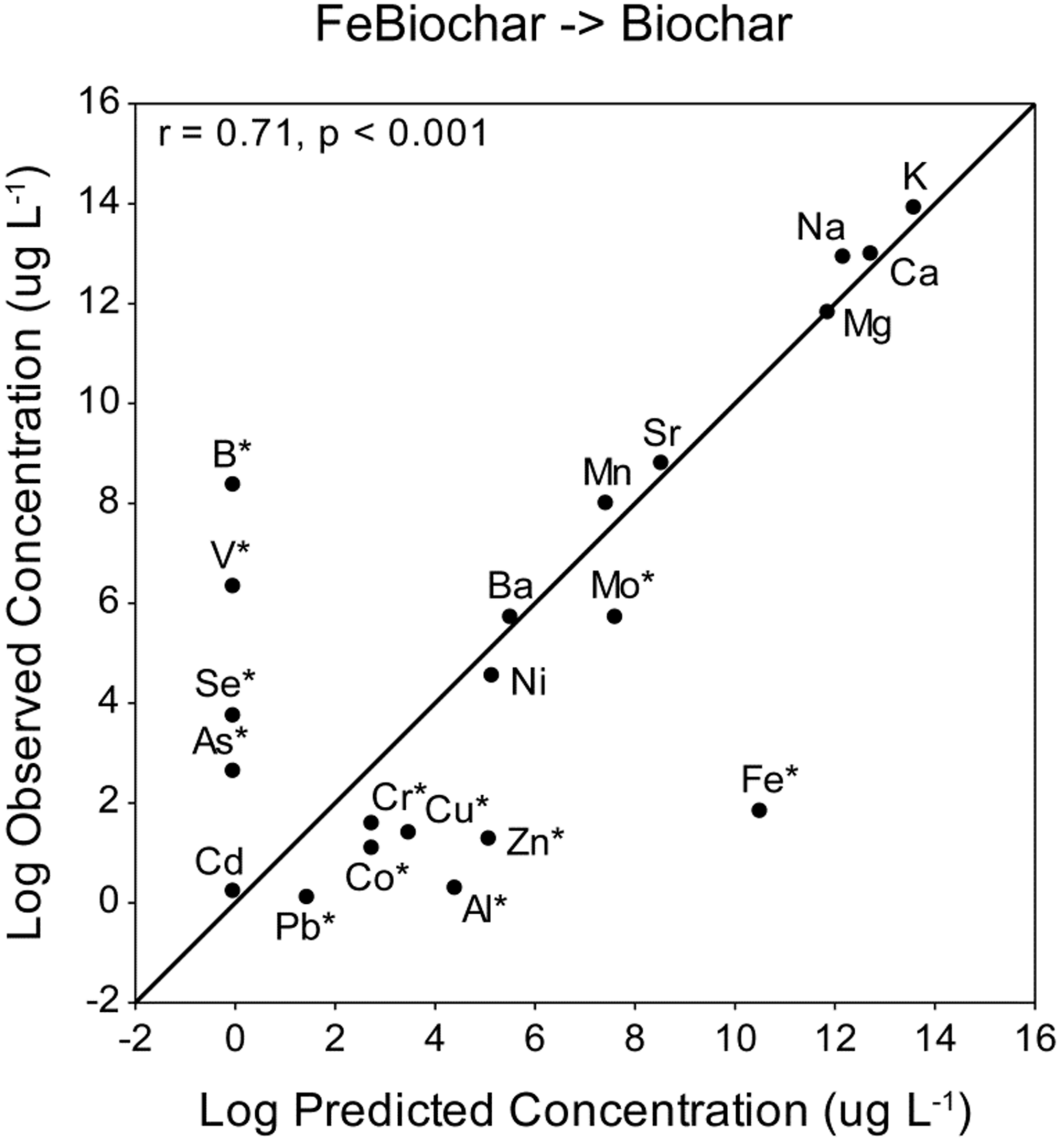
Scatterplot of predicted vs. observed concentrations of elements in ADW (µg L^−1^) following the “FeBC → BC” treatment. Data have been log transformed. The plotted line shows the expected relationship if all observed values are identical to predicted values. Points falling above the line were overestimated by the model (higher than expected final concentrations). Points falling below the line were underestimated by the model (lower than expected final concentrations). Asterisks indicate elements with a residual of greater than ±1.

In the “FeBC → BC” treatment 17 of the 21 elements were remediated close to, or below, predicted concentrations (Figure 6). Twelve elements out of 21 (Al, As, B, Co, Cr, Cu, Fe, Mo, Pb, Se, V & Zn) had residuals > ±1 (Figure 6). However, of these most were removed from ADW more effectively than was predicted by the model. As, B, Se and V had higher concentrations than predicted after the “FeBC → BC” treatment (Figure 6). Despite the high rate of leaching of Fe from the Fe-biochar treatment, the subsequent biochar treatment removed all of this Fe, resulting in a concentration (5 µg L^−1^) much lower than was predicted (39,451 µg L^−1^) (Figure 6; Table 2).

The predictive model is included as supporting information to this publication, allowing users to insert metal concentrations and desired effluents to see predicted treatment using the sequential approach (Table S3; instructions and assumptions available in the table caption). The elements are treated independently in the model with the assumption that the q-value for each element is independent of initial concentration. The model also assumes the effects of Fe-biochar and biochar use are additive. Therefore, the model output will show linear increases in the requisite Fe-biochar stocking densities as the initial concentration of each target metalloid in solution increases. The amount of biochar required in the second step of the sequential treatment will also increase linearly with the stocking density of Fe-biochar used. Instructions for use are supplied along with Table S3.

## Discussion

We have demonstrated that the sequential application of multiple biosorbents provides a comprehensive treatment of a complex effluent. The combination of targeted remediation of metalloids by Fe-biochar and metals by biochar resulted in a greater number of elements being treated compared to the application of either biosorbent independently. Furthermore, the reduction of metalloids followed by metals, by the sequential application of Fe-biochar then biochar, demonstrates that the effects of these biosorbents are additive. Our model predicted the concentration of most elements following treatments with Fe-biochar, although the model was slightly less effective at predicting the concentrations of elements when biochar was involved in the sequence. Regardless, the selectivity of the biosorbents was consistent and accurately modelled across the six water types (i.e. ADW treated by Fe-biochar, biochar, and combinations thereof) in which the initial element composition varied greatly. This provides the basis to predict the efficacy of these treatments (Fe-biochar and biochar in any order of application) in remediating the effluents from other industries.

Treatments that combined Fe-biochar and biochar (“FeBC → BC” and “FeBC + BC”) achieved the most comprehensive remediation of the greatest number of elements. However, sequential treatments with Fe-biochar (“FeBC → FeBC”) and biochar (“BC → BC”) were more effective at removing their target constituents (metalloids and metals, respectively). In the same way that the removal of a given element will be greater in isolation than when that element is present in a complex mixture, removing multiple elements from an effluent occurs at the expense of the sorption capacity of the biosorbent for each individual element [27]. Although treatment of a wastewater with a sequential application of macroalgal biosorbents has not been done previously, it has proven successful with other biosorbents. For example, in an analogous experiment, a sequential approach was taken to the treatment of wastewater using anaerobic fermented biological sludge, first using raw sludge to remove metals, then using sludge that had been pre-treated with a cation detergent (either Al^3+^ or Fe^3+^) to remove the metalloids [28]. We found no benefit in terms of efficacy for simultaneous application of the biosorbents, and some negatives regarding the loading ratios per unit volume, meaning water should be treated in two-step process of Fe-biochar followed by biochar.

Predicting the concentrations of elements following treatments of Fe-biochar and biochar with the model was very successful, with all of the sequential and single treatment scenarios resulting in at least a moderately strong correlation between predicted and observed values. Modelling has been used extensively in biosorption studies, however often only to predict and quantify the mechanism by which the element is being absorbed onto the biosorbent using Langmuir and Freundlich isotherms [12], [16], [29], [30]. Our empirical model performed particularly well when considering that the starting concentrations of most elements were significantly different to those in the effluent that was used to derive the model, and that the water quality after each treatment was unique, spanning 6 types of water treated with different combinations of biosorbents, resulting in a broad range of initial concentrations (Table 2). In this context, the model is as a robust tool for predicting the treatment of a broad range of waste water profiles by biosorbents derived from *Oedogonium*, and as a tool to develop working treatment strategies for a variety of waste water sources with known elemental compositions.

The model presented here intrinsically has some assumptions and limitations. The model is based on q-values from single deployment of the biosorbents in untreated ADW. The key assumption of the model is that there is a linear relationship between the extent of biosorption and biosorbent density that is independent of the initial concentration of each element in solution [16]. The model was slightly more effective at predicting final concentrations of elements after a single deployment of Fe-biochar or biochar than after two deployments. This suggests that the q-value is not entirely independent of the initial element concentrations because the biosorbents tend to remove slightly less of each element than predicted when initial concentrations are lower than the conditions under which the model was derived [31], [32]. However, the magnitude of difference between predicted and observed final concentrations are small despite the large variation in initial conditions across the six treatments. This reiterates the importance of assessing the efficacy of biosorbents under realistic initial conditions, rather than deriving models of biosorbent kinetics in single-element system with inordinately high initial metal concentrations. Regardless, our approach to determining q-values of biosorbents for multiple elements in a real-world effluent provides a robust metric for use in biosorption modelling and with additional testing under different scenarios, further accuracy and confidence limits may be applied.

### Conclusions

There is substantial literature on macroalgal biosorption but the majority of existing data has assessed elements in isolation, while an industrial effluent typically contains a multitude of co-existing, interacting elements. Given the large quantities of biomass that will inevitably be required to treat industrial effluents, it will also be necessary to select and cultivate biomass locally for the express purposes of bioremediation. We have demonstrated that different types of biosorbents (Fe-biochar and biochar) can be produced from a single feedstock that is native to an industrial facility (the freshwater macroalga *Oedogonium*, see also [18], [33]) and used to sequentially treat metalloids and metals from a complex effluent. Through the use of a predictive model we demonstrate that sequential biosorption is largely predictable and consistent across a wide range of initial conditions. Targeting metalloids that do not have a natural affinity for biochar requires Fe-treatment, which inherently results in a suite of additional metals being released into solution. This metal leaching can in turn be addressed through the use of biochar as a final treatment. Therefore, while biosorption has been widely cited as a sustainable and cost-effective means of treating waste waters, our data clearly show that the reality is far more complex than is typically acknowledged, but remains achievable.

## Supporting Information

Figure S1 **Change in solution concentration of (a) potassium, (b) manganese, and (c) vanadium following sequential exposure to Fe-biochar and biochar.** Sequential exposure of the ADW to biochar, Fe-biochar, and Fe-biochar followed by biochar and represented as dotted, dashed and solid lines, respectively. Exposure of ADW to both Fe-biochar and biochar simultaneously is represented as a round dot. Simultaneous exposure only had one application yet is placed under treatment 2 to compare with the final concentrations of the other treatments. ANZECC trigger level represented by a horizontal grey line. Error bars show standard error.
(DOCX)

Table S1 **Change in dissolved elemental concentration (µg L^−1^) in ADW following treatment with biochar and Fe-biochar for 1 h at a solution pH of 7.1, and the derived q-values (µg g^−1^).** Data were collected during experiments for Kidgell et al (in press).
(DOCX)

Table S2 **Summary table for ANOVA tests run on each of the 21 elements investigated.** One-way analysis of variance tests were run on final elemental concentration with the factor of Treatment. Type III sum of squares was used. All tests met the assumption of homogeneity of variance, normality of residuals and independence. Transformation of the data were required for some elements, the transformation applied is listed next to the title. Factors in bold indicate significance under alpha of 0.05.
(DOCX)

Table S3 **Predictive biosorption model for treatment of industrial waste water through sequential Fe-biochar and Biochar applications.** ***Instructions to users*** **.** 1. Cells that can be edited by the user are shaded orange. 2. Input initial dissolved concentrations of elements and the volume of effluent requiring treatment in the orange shaded cells at Step 1. The model will calculate the recommended Fe-biochar stocking density which is displayed at Step 2. 3. Select the maximum Fe-biochar stocking density for elements of interest from Step 2 and enter it in the orange shaded cell at Step 3. The model will calculate the stocking density of biochar required to remove leached and existing metals in the effluent after Fe-biochar deployment which is played at Step 4. 4. Select the maximum biochar stocking density for elements of interest from Step 4 and enter it in the orange shaded cell at Step 5. The model will calculate the predicted final concentrations of metalloids and metals after the sequential application of Fe-biochar and biochar in the effluent at Step 6.
(XLSX)

## References

[1] Volesky B (1999) Biosorption for the next century. In: Amils R, Ballester A, editors. Biohydrometallurgy and the environment: Toward the mining of the 21st century. Amsterdam: Elsevier. pp. 161–170.

[2] Frankenberger WT Jr, Amrhein C, Fan TWM, Flaschi D, Glater J, et al (2004) Advanced treatment technologies in the remediation of seleniferous drainage waters and sediments. Irrig Drain Syst 18: 19–42.

[3] Hamilton SJ (2004) Review of selenium toxicity in the aquatic food chain. Sci Total Environ 326: 1–31. 15142762 10.1016/j.scitotenv.2004.01.019

[4] Sappington KG (2002) Development of aquatic life criteria for selenium: a regulatory perspective on critical issues and research needs. Aquat Toxicol 57: 101–113. 11879941 10.1016/s0166-445x(01)00267-3

[5] Davis TA, Volesky B, Mucci A (2003) A review of the biochemistry of heavy metal biosorption by brown algae. Water Res 37: 4311–4330. 14511701 10.1016/S0043-1354(03)00293-8

[6] Gadd GM (2009) Biosorption: critical review of scientific rationale, environmental importance and significance for pollution treatment. J Chem Technol Biotechnol 84: 13–28.

[7] Volesky B (2007) Biosorption and me. Water Res 41: 4017–4029. 17632204 10.1016/j.watres.2007.05.062

[8] Saunders RJ, Paul NA, Hu Y, de Nys R (2012) Sustainable sources of biomass for bioremediation of heavy metals in waste water derived from coal-fired power generation. PLOS One 7: e36470. 22590550 10.1371/journal.pone.0036470 PMC3348934

[9] Esmaeili A, Ghasemi S, Sohrabipour J (2010) Biosorption of copper from wastewater by activated carbon preparation from alga *Sargassum* sp. Nat Prod Res 24: 341–348. 20221940 10.1080/14786410903064915

[10] Pavasant P, Apiratikul R, Sungkhum V, Suthiparinyanont P, Wattanachira S, et al (2006) Biosorption of Cu^2+^, Cd^2+^, Pb^2+^, and Zn^2+^ using dried marine green macroalga *Caulerpa lentillifera* . Bioresour Technol 97: 2321–2329. 16330209 10.1016/j.biortech.2005.10.032

[11] Sheng PX, Ting YP, Chen JP, Hong L (2004) Sorption of lead, copper, cadmium, zinc, and nickel by marine algal biomass: characterization of biosorptive capacity and investigation of mechanisms. J Colloid Interface Sci 275: 131–141. 15158390 10.1016/j.jcis.2004.01.036

[12] Moghaddam MR, Fatemi S, Keshtkar A (2013) Adsorption of lead (Pb^2+^) and uranium (UO_2_ ^2+^) cations by brown algae; experimental and thermodynamic modeling. Chem Eng J 231: 294–303.

[13] Sulaymon AH, Mohammed AA, Al-Musawi TJ (2013) Competitive biosorption of lead, cadmium, copper, and arsenic ions using algae. Environ Sci Pollut Res Int 20: 3011–3023. 23054774 10.1007/s11356-012-1208-2 PMC3633787

[14] Kidgell JT, de Nys R, Hu Y, Paul NA, Roberts DA (2014) Bioremediation of a complex industrial effluent by biosorbents derived from freshwater macroalgae. PLOS One. In press. 10.1371/journal.pone.0094706 PMC4053327 24919058

[15] Fomina M, Gadd GM (2014) Biosorption: current perspectives on concept, definition and application. Bioresour Technol. Accepted 03.01.2014. 10.1016/j.biortech.2013.12.102 24468322

[16] Mehta SK, Gaur JP (2005) Use of algae for removing heavy metal ions from wastewater: Progress and prospects. Crit Rev Biotechnol 25: 113–152. 16294830 10.1080/07388550500248571

[17] Naja G, Volesky B (2011) The mechanism of metal cation and anion biosorption. In: Kotrba P, Mackova M, Macek T, editors. Chemistry and ecology. Amsterdam: Springer Netherlands. pp. 19–58.

[18] Gupta VK, Rastogi A (2008) Equilibrium and kinetic modelling of cadmium(II) biosorption by nonliving algal biomass *Oedogonium* sp. from aqueous phase. J Hazard Mater 153: 759–766. 17942222 10.1016/j.jhazmat.2007.09.021

[19] Murphy V, Hughes H, McLoughlin P (2008) Comparative study of chromium biosorption by red, green and brown seaweed biomass. Chemosphere 70: 1128–1134. 17884133 10.1016/j.chemosphere.2007.08.015

[20] Kratochvil D, Pimentel P, Volesky B (1998) Removal of trivalent and hexavalent chromium by seaweed biosorbent. Environ Sci Technol 32: 2693–2698.

[21] Deng L, Zhang Y, Qin J, Wang X, Zhu X (2009) Biosorption of Cr(VI) from aqueous solutions by nonliving green algae *Cladophora albida* . Miner Eng 22: 372–377.

[22] Roberts DA, Paul NA, Dworjanyn SA, Hu Y, Bird MI, et al. (2014) *Gracilaria* waste biomass (sampah rumput laut) as a bioresource for selenium biosorption. J Appl Phycol. Accepted 09.05.2014.

[23] Lawton RJ, de Nys R, Skinner S, Paul NA (2014) Isolation and selection of *Oedogonium* species and strains for biomass applications. PLOS One 9: e90223. 24603705 10.1371/journal.pone.0090223 PMC3946159

[24] Bird MI, Wurster CM, de Paula Silva PH, Bass AM, de Nys R (2011) Algal biochar—production and properties. Bioresour Technol 102: 1886–1891. 20797850 10.1016/j.biortech.2010.07.106

[25] Quinn GP, Keough MJ (2002) Experimental design and data analysis for biologists. Cambridge: Cambridge University Press. 556 p.

[26] ANZECC (2000) An introduction to the Australian and New Zealand guidelines for fresh and marine water quality. Canberra: Australian and New Zealand Environment and Conservation Council. 314 p.

[27] Figueira MM, Volesky B, Ciminelli VS (1997) Assessment of interference in biosorption of a heavy metal. Biotechnol Bioeng 54: 344–350. 18634101 10.1002/(SICI)1097-0290(19970520)54:4<344::AID-BIT7>3.0.CO;2-K

[28] Geuens L (1987) Waterwater purification process. Patent US 06/901,649.

[29] Ali I, Gupta VK (2006) Advances in water treatment by adsorption technology. Nat Protoc 1: 2661–2667. 17406522 10.1038/nprot.2006.370

[30] Yang JB, Volesky B (1999) Removal and concentration of uranium by seaweed biosorbent. In: Amils R, Ballester A, editors. Biohydrometallurgy and the environment. Toward the mining of the 21st Century. Amsterdam:Elsevier. pp. 483–492.

[31] Plazinski W (2013) Binding of heavy metals by algal biosorbents. Theoretical models of kinetics, equilibria and thermodynamics. Adv Colloid Interface Sci 197: 58–67. 23688631 10.1016/j.cis.2013.04.002

[32] Plazinski W (2013) Equilibrium and kinetic modeling of metal ion biosorption: on the ways of model generalization for the case of multicomponent systems. Adsorption 19: 659–666.

[33] Bakatula E, Cukrowska E, Weiersbye I, Mihaly-Cozmuta L, Peter A, et al. (2014) Biosorption of trace elements from aqueous systems in gold mining sites by the filamentous green algae (*Oedogonium* sp.). J Geochemical Explor. In press.

