# Correction: The Sequential Application of Macroalgal Biosorbents for the Bioremediation of a Complex Industrial Effluent

**DOI:** 10.1371/journal.pone.0106375

**Published:** 2014-08-18

**Authors:** The PLOS ONE Staff

## Notice of Republication

This article was republished on August 5, 2014, to replace the file for Table S3. Please view this article again to download the correct file.
