# Supplementary material for: The Sequential Application of Macroalgal Biosorbents for the Bioremediation of a Complex Industrial Effluent
Source: PLoS One. 2014 Jul 25;9(7):e101309. doi: 10.1371/journal.pone.0101309 (PMC4111303; doi:10.1371/journal.pone.0101309)
Supplement: Figure S1 — Change in solution concentration of (a) potassium, (b) manganese, and (c) vanadium following sequential exposure to Fe-biochar and biochar. Sequential exposure of the ADW to biochar, Fe-biochar, and Fe-biochar followed by biochar and represented as dotted, dashed and solid lines, respectively. Exposure of ADW to both Fe-biochar and biochar simultaneously is represented as a round dot. Simultaneous exposure only had one application yet is placed under treatment 2 to compare with the final concentrations of the other treatments. ANZECC trigger level represented by a horizontal grey line. Error bars show standard error. (DOCX) [file pone.0101309.s001.docx]

**

**

**Figure S1** Change in solution concentration of (a) potassium, (b) manganese, and (c) vanadium following sequential exposure to Fe-biochar and biochar. Sequential exposure of the ADW to biochar, Fe-biochar, and Fe-biochar followed by biochar and represented as dotted, dashed and solid lines, respectively. Exposure of ADW to both Fe-biochar and biochar simultaneously is represented as a round dot. Simultaneous exposure only had one application yet is placed under treatment 2 to compare with the final concentrations of the other treatments. ANZECC trigger level represented by a horizontal grey line. Error bars show standard error.
