# Supplementary material for: The Sequential Application of Macroalgal Biosorbents for the Bioremediation of a Complex Industrial Effluent
Source: PLoS One. 2014 Jul 25;9(7):e101309. doi: 10.1371/journal.pone.0101309 (PMC4111303; doi:10.1371/journal.pone.0101309)
Supplement: Table S1 — Change in dissolved elemental concentration (µg L−1) in ADW following treatment with biochar and Fe-biochar for 1 h at a solution pH of 7.1, and the derived q-values (µg g−1). Data were collected during experiments for Kidgell et al (in press). (DOCX) [file pone.0101309.s002.docx]

**Table S1** Change in dissolved elemental concentration (µg L^-1^) in ADW following treatment with biochar and Fe-biochar for 1 h at a solution pH of 7.1, and the derived q-values (µg g^-1^). Data were collected during experiments for Kidgell et al (under review).

| Element | Initial Concentration [µg L^-1^ (± SE)] | | Final Concentration [µg L^-1^ (± SE)] | | | | q-value (µg g^-1^) | |
| --- | --- | --- | --- | --- | --- | --- | --- | --- |
|  |  |  | Biochar | | Fe-Biochar | | Biochar | Fe-Biochar |
| Al | 114 | (29) | 40 | (18) | 432 | (135) | 7.4 | -31.8 |
| As | 43 | (5.5) | 40 | (4.8) | 16 | (1.8) | 0.3 | 2.7 |
| B | 7530 | (896) | 6767 | (1000) | 6797 | (994) | 76.3 | 73.3 |
| Ba | 108 | (2.3) | 114 | (2.8) | 191 | (6.2) | -0.6 | -8.3 |
| Ca | 330500 | (1528) | 330000 | (8888) | 355667 | (8762) | 50 | -2517 |
| Cd | 2.3 | (0.2) | 1.0 | (0.2) | 1.6 | (0.1) | 0.13 | 0.07 |
| Co | 0.6 | (0.2) | 0.3 | (0.1) | 12.2 | (3.1) | 0.03 | -1.2 |
| Cr | 5.6 | (3.5) | 4.5 | (3.0) | 21 | (5.8) | 0.1 | -1.5 |
| Cu | 1.9 | (0.9) | 1.9 | (1.0) | 24 | (5.8) | 0.00 | -2.2 |
| Fe | 1401 | (360) | 675 | (215) | 33533 | (6786) | 72.6 | -3213 |
| K | 30022 | (11416) | 163067 | (59653) | 28220 | (11708) | -13305 | 180 |
| Mg | 93700 | (301) | 95967 | (617) | 12333 | (2603) | -226.7 | 8137 |
| Mn | 3 | (0.7) | 67 | (6.6) | 1001 | (131) | -6.4 | -99.8 |
| Mo | 1437 | (167) | 1397 | (122) | 253 | (26) | 4.0 | 118 |
| Na | 446000 | (2363) | 405333 | (5365) | 443667 | (4910) | 4067 | 233 |
| Ni | 53 | (7.4) | 20 | (3.7) | 298 | (59) | 3.3 | -24.5 |
| Pb | 0.26 | (0.1) | 0.1 | (0.08) | 3 | (0.1) | 0.02 | -0.3 |
| Se | 81 | (4.1) | 76 | (1.9) | 23 | (3.4) | 0.5 | 5.8 |
| Sr | 1655 | (272) | 1790 | (260) | 1847 | (262) | -13.5 | -19.2 |
| V | 1098 | (102) | 945 | (71) | 963 | (80) | 15.3 | 13.5 |
| Zn | 64 | (11) | 6.7 | (4.2) | 409 | (75) | 5.7 | -34.5 |

- All treatments were conducted with 60 ml of effluent and 0.6 g of biosorbent
