# Supplementary material for: The Sequential Application of Macroalgal Biosorbents for the Bioremediation of a Complex Industrial Effluent
Source: PLoS One. 2014 Jul 25;9(7):e101309. doi: 10.1371/journal.pone.0101309 (PMC4111303; doi:10.1371/journal.pone.0101309)
Supplement: Table S2 — Summary table for ANOVA tests run on each of the 21 elements investigated. One-way analysis of variance tests were run on final elemental concentration with the factor of Treatment. Type III sum of squares was used. All tests met the assumption of homogeneity of variance, normality of residuals and independence. Transformation of the data were required for some elements, the transformation applied is listed next to the title. Factors in bold indicate significance under alpha of 0.05. (DOCX) [file pone.0101309.s003.docx]

**Table S2** Summary table for ANOVA tests run on each of the 21 elements investigated. One-way analysis of variance tests were run on final elemental concentration with the factor of Treatment. Type III sum of squares was used. All tests met the assumption of homogeneity of variance, normality of residuals and independence. Transformation of the data were required for some elements, the transformation applied is listed next to the title. Factors in bold indicate significance under alpha of 0.05.

| Element | Factor | DF | SS | MS | F | P |
| --- | --- | --- | --- | --- | --- | --- |
| Aluminium | **Treatment** | **3** | **1.6E+6** | **543,864** | **1823** | **<0.001** |
|  | Error | 8 | 2387 | 298 |  |  |
| Arsenic | **Treatment** | **3** | **680** | **227** | **734** | **<0.001** |
|  | Error | 8 | 2.47 | 0.31 |  |  |
| Boron | **Treatment** | **3** | **1.0E+6** | **337,089** | **176** | **<0.001** |
|  | Error | 8 | 15333 | 1917 |  |  |
| Cadmium | **Treatment** | **3** | **3.60** | **1.20** | **3605** | **<0.001** |
|  | Error | 8 | 0.00 | 0.00 |  |  |
| [Log] Chromium | **Treatment** | **3** | **22.1** | **7.36** | **199** | **<0.001** |
|  | Error | 8 | 0.30 | 0.04 |  |  |
| [Forthroot] Copper | **Treatment** | **3** | **3.85** | **1.28** | **1828** | **<0.001** |
|  | Error | 8 | 0.01 | 0.00 |  |  |
| Manganese | **Treatment** | **3** | **3.9E+7** | **1.3E+7** | **230** | **<0.001** |
|  | Error | 8 | 454,733 | 56,842 |  |  |
| [Squareroot] Molybdenum | **Treatment** | **3** | **7129** | **2376** | **7676** | **<0.001** |
|  | Error | 8 | 2.48 | 0.31 |  |  |
| [Log] Nickel | **Treatment** | **3** | **45.4** | **15.1** | **1071** | **<0.001** |
|  | Error | 8 | 0.11 | 0.01 |  |  |
| Lead | **Treatment** | **3** | **101** | **33.6** | **4482** | **<0.001** |
|  | Error | 8 | 0.06 | 0.01 |  |  |
| Selenium | **Treatment** | **3** | **13,375** | **4458** | **2314** | **<0.001** |
|  | Error | 8 | 15.4 | 1.93 |  |  |
| [Squareroot] Zinc | **Treatment** | **3** | **4180** | **1393** | **8098** | **<0.001** |
|  | Error | 8 | 1.38 | 0.17 |  |  |
